# Association between periodontitis and cardiometabolic index (CMI): a study from NHANES 2009–2014

**DOI:** 10.1038/s41598-024-78382-7

**Published:** 2024-11-18

**Authors:** Li Shuning, Zhang Zhiyong, Yang Wei, Liu Jilun, Fan Xuhui

**Affiliations:** The Second Hospital of Hebei Medical University, Shijiazhuang, 050000 Hebei China

**Keywords:** NHANES, Cardiometabolic index (CMI), Periodontitis, Cross-sectional study, Dentistry, Health policy, Risk factors

## Abstract

Cardiometabolic index (CMI) is a novel anthropometric metric that integrates lipid and adiposity characteristics. The correlation between periodontitis development and CMI is ambiguous. The objective of this study was to establish the association between CMI and periodontitis by analyzing data from the NHANES (National Health and Nutrition Examination Survey) database. A cross-sectional study was conducted on a cohort of 6188 people selected from the NHANES database, covering the period from 2009 to 2014. The study employed multivariate logistic regression to examine the independent correlation between CMI and periodontitis. Subgroup data were analyzed and interaction tests were conducted to assess the impact of variables on the correlation between CMI and periodontitis. The CMI index was significantly and positively associated with the presence of periodontitis (β = 0.03, 95%CI(0.01, 0.05), *p* = 0.0092). In addition, a U-shaped relationship was found between CMI index and periodontitis severity in an older American population (65 < = age < = 80, with a folding point of 1.44, *p* = 0.008). This study demonstrates a significant correlation between CMI and periodontitis, positioning CMI as a crucial indicator for assessing periodontal health. Future efforts should prioritize oral hygiene interventions for patients with elevated CMI levels to facilitate early intervention and enhance overall health outcomes.

## Introduction

Periodontitis is a chronic non-communicable disease (NCD) that affects 11.2% of the worldwide population and is the sixth most widespread human illness. Its rates of prevalence range from 45 to 50% over the whole population^1^. There is substantial evidence indicating a direct connection between periodontitis and other systemic illnesses^2–5^. Severe periodontitis has a strong correlation with both all-cause and cardiovascular mortality, regardless of other contributing variables^5,6^. According to these studies, there is a direct connection between periodontitis and a wide variety of metabolic problems.

The waist-to-height ratio (WHtR) to high-density lipoprotein cholesterol (HDL-C) ratio^7^ is multiplied to determine the CMI, which was introduced in 2015. Obesity and lipid indicators, such as body mass index (BMI), waist circumference (WC), triglycerides (TG), and body mass index (BAI), are less effective in predicting metabolic disorders compared to CMI^7–9^.

The CMI is a more effective predictor of metabolic disorders as it integrates both the waist-to-height ratio (WHtR) and high-density lipoprotein cholesterol (HDL-C) levels, providing a comprehensive assessment of an individual’s metabolic health, particularly regarding the impact of abdominal fat distribution and the protective role of cholesterol, thereby outperforming traditional obesity and lipid indices.

Given the favorable diagnostic potential of CMI for a range of metabolic diseases. Furthermore, there exists a complex and inseparable connection between periodontitis and several systemic disorders. Hence, it is imperative to do more research on the possible contribution of CMI in the diagnosis of periodontitis, as no relevant studies have been documented thus far.

This study aims to investigate the potential role of the CMI in the assessment of periodontitis, utilizing data from the NHANES database.

## Methods

### Data sources and study population

The National Health and Nutrition Examination Survey (NHANES) gathered information on factors that people were exposed to and the resulting outcomes. This data was collected throughout three consecutive cycles from 2009 to 2014. You may find more information at https://www.cdc.gov/nchs/nhanes. The National Centre for Health Statistics (NCHS) is in charge of NHANES administration. The data were collected through health interviews conducted at participants’ residences, health checks carried out at mobile testing centres (MECs), and laboratory samples. There was no need for a supplementary ethical review for this manuscript, as NHANES underwent an ethical review by the National Ethical Review Board for Research in Health Statistics^10^.

**Fig. 1:**
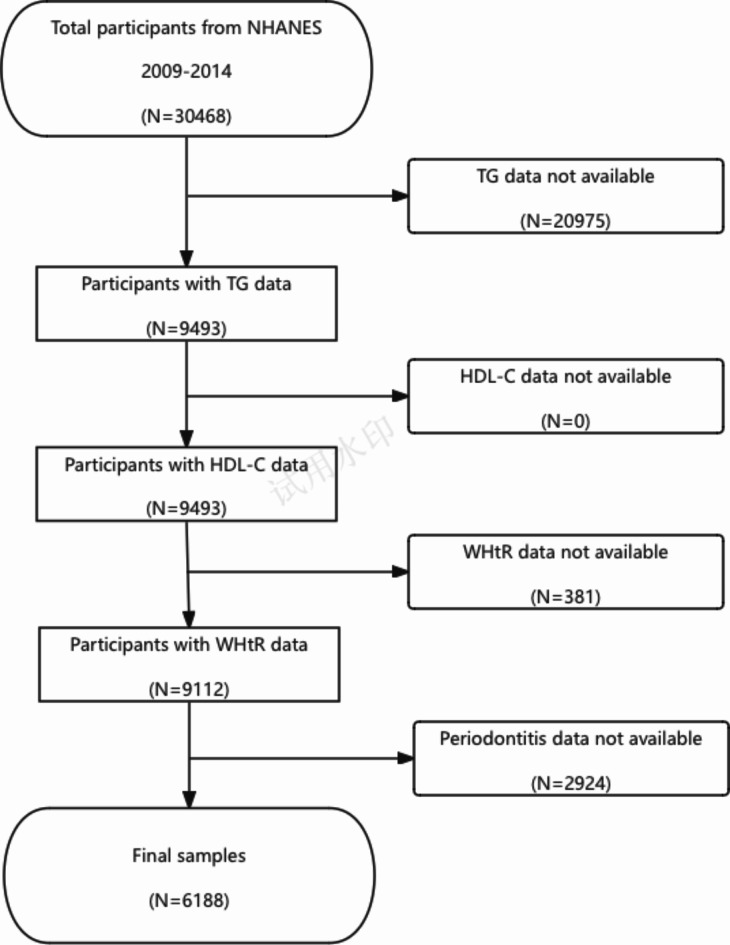
Flowchart of participant selection. NHANES, National Health and Nutrition Examination Survey; TG, triglycerides; HDL-C, high-density lipoprotein cholesterol; WHtR, waist-to-height ratio.

The study, which initially comprised 30,468 participants (NHANES 2009 through 2014), incorporated the most recent periodontal examination data for US adults. The eligibility of a subject to participate was determined based on the following criteria: (1) NHANES participants aged thirty years or older; (2) individuals who had undergone an oral periodontal examination; and (3) NHANES participants with available data on their waist-to-height ratio, triglycerides, and HDL-C. In the end, a grand total of 6188 individuals were enrolled in the research. Figure 1 depicts the procedure of data filtration.

### Outcome variable

Clinical attachment loss (AL) and periodontal pocket probing depth (PD) are typical indicators for evaluating the severity of periodontitis^11^. The periodontist examined the 28 eligible participants’ teeth at the mobile examination center, exploring each tooth at six sites, but did not include the third molars^12^. This study employed the CDC/AAP classification criteria for periodontitis, which were created by Eke et al. in 2012^13^. 2 neighboring AL ≥ 3 mm, ≥ 2 neighboring PD ≥ 4 mm (not on the same tooth), or 1 neighboring PD ≥ 5 mm were the criteria for mild periodontitis. A periodontitis that was classified as moderate was defined as two adjacent alveoli (AL) that were either ≥ 4 mm (not on the same tooth) or ≥ 2 mm (in the same tooth). Two neighboring sites AL ≥ 6 mm (not on the same tooth) and at least one neighboring site PD ≥ 5 mm were used to designate severe periodontitis. As per the aforementioned criteria, individuals are classified as having mild periodontitis, moderate periodontitis, severe periodontitis, or no periodontitis.

### Exposure variable

Cardiometabolic Index was determined by employing the subsequent formula: CMI = TG(mmol/L)/HDL-C(mmol/L)*WHtR. The data was categorized into four categories based on quartiles: CMIQ1 (CMI less than 0.311), CMIQ2 (CMI between 0.311 and 0.534), CMIQ3 (CMI between 0.534 and 0.938), CMIQ4 (CMI more than or equal to 0.938).

### Confounding variables

Covariates were chosen in accordance with clinical expertise and prior research. The missing data were imputed using multiple imputation. This study included the following covariates: gender, age, race, alcohol consumption history, smoking history, diabetes, glycohemoglobin, hypertension, heart disease, hypercholesterolemia, and cancer. Gender was categorized as male or female, and age was segmented into four categories to investigate the impact of CMI on periodontal disease across different age groups. The race categories included Mexican American, Other Hispanic, Non-Hispanic White, Non-Hispanic Black, and Other Race. Participants were classified into four groups based on their frequency of alcohol consumption in the past 12 months: no alcohol consumption (< 1), light drinking ( > = 1,<10), moderate drinking ( > = 10, < 20), and heavy drinking ( > = 20). Participants were divided into two groups based on whether they had smoked more than 100 cigarettes: YES or NO. Diabetes status was categorized into three groups: YES, NO, or Borderline, based on whether a doctor had diagnosed the participant with diabetes. Hypertension status was divided into two groups based on whether the participant had been told they had high blood pressure: YES or NO. Heart disease status was categorized into two groups based on whether the participant had been told they had coronary heart disease: YES or NO. Hypercholesterolemia status was split into two groups based on whether a doctor had diagnosed the participant with high cholesterol level: YES or NO. Cancer status was categorized into two groups based on whether the participant had been told they had cancer or malignancy: YES or NO.

### Statistical analysis

The statistical analysis of the results was performed using the R software (R4.2.1, http://www.R-project.org) in combination with Empower Stats 4.1, which could be accessed at http://www.empowerstats.com. The Kruskal-Wallis H-test was used to evaluate statistical disparities among different CMI groups (quartiles) for continuous variables, while the chi-square test was performed for categorical variables. The study utilized multivariate logistic regression to investigate the independent association between CMI and periodontitis. Three models were employed: an unadjusted model, a model adjusted for age, gender, and race (model 1), and a model adjusted for age, gender, race, alcohol consumption, smoking, diabetes, hypertension, heart disease, hypercholesterolemia, and cancer (model 2). The robustness of the results was guaranteed by employing trend p values as sensitivity analyses^14^. Analyzed subgroups and conducted interaction tests to assess the influence of variables on the relationship between CMI and periodontitis^15^. In the end, we performed smoothed curve fitting and threshold effect experiments to examine the association between the CMI and periodontitis, as well as any potential nonlinear relationships.

## Results

### Baseline characteristics of the population

The Table 1 displays the fundamental features of the research population. The analysis covered a total of 6188 individuals. The average CMI was 0.81. The study classified CMI into four groups: CMIQ1 (CMI < 0.311), CMIQ2 (0.311 ≤ CMI < 0.534), CMIQ3 (0.534 ≤ CMI < 0.938), and CMIQ4 (CMI ≥ 0.938). The participants had an average age of 53.98 years, with 48.32% of them being male and up to 45.12% being Non-Hispanic White. Out of the sample, 3497 individuals, which represents 56.51% of the total, were not diagnosed with periodontitis. On the other hand, 161 patients (2.60%), 2153 patients (34.79%), and 377 patients (6.09%) had mild, moderate, and severe periodontitis, respectively.

**Table 1:** Characterization of the study population based on cardiometabolic index quartiles.

Variables	CMIQ1	CMIQ2	CMIQ3	CMIQ4	*P*-value
N	1547	1547	1547	1547	
Age	52.47 ± 14.87	54.54 ± 14.98	55.17 ± 14.89	53.76 ± 14.20	< 0.001
Glycohemoglobin	5.52 ± 0.65	5.75 ± 0.98	5.91 ± 1.09	6.22 ± 1.40	< 0.001
Gender					< 0.001
male	563 (36.39%)	734 (47.45%)	779 (50.36%)	914 (59.08%)	
female	984 (63.61%)	813 (52.55%)	768 (49.64%)	633 (40.92%)	
Race					< 0.001
Mexican American	126 (8.14%)	193 (12.48%)	257 (16.61%)	290 (18.75%)	
Other Hispanic	121 (7.82%)	150 (9.70%)	176 (11.38%)	195 (12.61%)	
Non-Hispanic White	692 (44.73%)	664 (42.92%)	663 (42.86%)	773 (49.97%)	
Non-Hispanic Black	421 (27.21%)	348 (22.50%)	274 (17.71%)	146 (9.44%)	
Other Race	187 (12.09%)	192 (12.41%)	177 (11.44%)	143 (9.24%)	
Alcohol Consumption					< 0.001
< 1	197 (12.73%)	250 (16.16%)	312 (20.17%)	338 (21.85%)	
>=1, < 10	1287 (83.19%)	1236 (79.90%)	1163 (75.18%)	1142 (73.82%)	
>=10, < 20	41 (2.65%)	36 (2.33%)	46 (2.97%)	42 (2.71%)	
>=20	22 (1.42%)	25 (1.62%)	26 (1.68%)	25 (1.62%)	
Smoking					< 0.001
YES	633 (40.92%)	649 (41.95%)	717 (46.35%)	825 (53.33%)	
NO	914 (59.08%)	898 (58.05%)	830 (53.65%)	722 (46.67%)	
Diabetes					< 0.001
YES	81 (5.24%)	177 (11.44%)	267 (17.26%)	326 (21.07%)	
NO	1432 (92.57%)	1325 (85.65%)	1246 (80.54%)	1161 (75.05%)	
Borderline	34 (2.20%)	45 (2.91%)	34 (2.20%)	60 (3.88%)	
Hypertension					< 0.001
YES	472 (30.51%)	631 (40.79%)	687 (44.41%)	799 (51.65%)	
NO	1075 (69.49%)	916 (59.21%)	860 (55.59%)	748 (48.35%)	
Heart Disease					< 0.001
YES	46 (2.97%)	62 (4.01%)	82 (5.30%)	95 (6.14%)	
NO	1501 (97.03%)	1485 (95.99%)	1465 (94.70%)	1452 (93.86%)	
Hypercholesterolemia					< 0.001
YES	400 (25.86%)	595 (38.46%)	645 (41.69%)	734 (47.45%)	
NO	1147 (74.14%)	952 (61.54%)	902 (58.31%)	813 (52.55%)	
Cancer					0.697
YES	170 (10.99%)	158 (10.21%)	153 (9.89%)	169 (10.92%)	
NO	1377 (89.01%)	1389 (89.79%)	1394 (90.11%)	1378 (89.08%)	
Periodontitis					0.01
No periodontitis	923 (59.66%)	868 (56.11%)	877 (56.69%)	829 (53.59%)	
Mild periodontitis	43 (2.78%)	32 (2.07%)	45 (2.91%)	41 (2.65%)	
moderate periodontitis	510 (32.97%)	556 (35.94%)	519 (33.55%)	568 (36.72%)	
Severe periodontitis	71 (4.59%)	91 (5.88%)	106 (6.85%)	109 (7.05%)	

Results in table: Mean ± SD / N(%).

### Relationship between CMI index and periodontitis

In our study, we found a significant association between CMI and the risk of periodontitis(Table 2). In the unadjusted model, an increase in CMI was associated with an increased risk of periodontitis (β = 0.04, 95%CI: 1.02–1.06, *P* = 0.0004). This association remained significant even after adjusting for potential confounders such as age, gender, race, lifestyle factors, and chronic diseases (Model 1: β = 0.03, 95%CI: 1.01–1.06, *P* = 0.0022; Model 2: β = 0.03, 95%CI: 1.01–1.05, *P* = 0.0092), suggesting that CMI may be an independent predictor of periodontitis.

**Table 2:** Relationship between CMI index and periodontitis.

Exposure	Unadjusted model	Model 1	Model 2
CMI	0.04 (0.02, 0.06) 0.0004	0.03 (0.01, 0.06) 0.0022	0.03 (0.01, 0.05) 0.0092

Results in table: β (95%CI) P-value.

Outcome variable: Periodontitis.

Exposure variable: CMI.

unadjusted model adjusts for: None.

model 1 adjusts for age, gender, race.

model 2 adjusts for age, gender, race, alcohol consumption, smoking, diabetes, glycohemoglobin, hypertension, heart disease, hypercholesterolemia, and cancer.

In the interaction test, we developed the age groups (AGEQ1 30 < = age < 41, AGEQ2 41 < = age < 53, AGEQ3 53 < = age < 65, AGEQ4 65 < = age < = 80) by four. The models were adjusted for gender, race, alcohol consumption, smoking, diabetes, glycohemoglobin, hypertension, heart disease, hypercholesterolemia, and cancer. Age was considered as the effect modifier. The results indicate that as age increases, the impact of increased CMI on the presence of periodontitis becomes more significant. The P-value for interaction is 0.00028, suggesting that age significantly modifies the relationship between CMI and periodontitis. The shift resulting from gender did not have a significant impact, as indicated by a P-value of 0.276 for the interaction (Table 3).

**Table 3:** A test of the interaction between CMI and periodontitis.

Model II*				
Exposure: CMI	Effect modifier: Age	Statistics		
		N	β(95% CI)	P-values
CMIQ1	AGEQ1	413	Ref.	
CMIQ2	AGEQ1	339	-0.03 (-0.18, 0.12)	0.7137
CMIQ3	AGEQ1	327	-0.10 (-0.25, 0.06)	0.2188
CMIQ4	AGEQ1	339	-0.00 (-0.16, 0.16)	0.9731
CMIQ1	AGEQ2	426	-0.59 (-1.88, 0.71)	0.3745
CMIQ2	AGEQ2	392	-0.51 (-1.80, 0.79)	0.4422
CMIQ3	AGEQ2	367	-0.39 (-1.68, 0.91)	0.5585
CMIQ4	AGEQ2	413	-0.40 (-1.69, 0.89)	0.5443
CMIQ1	AGEQ3	333	0.33 (-0.92, 1.58)	0.6058
CMIQ2	AGEQ3	382	0.46 (-0.79, 1.70)	0.4732
CMIQ3	AGEQ3	386	0.35 (-0.90, 1.59)	0.5867
CMIQ4	AGEQ3	408	0.46 (-0.78, 1.71)	0.4657
CMIQ1	AGEQ4	375	0.03 (-1.20, 1.25)	0.9665
CMIQ2	AGEQ4	434	-0.06 (-1.28, 1.16)	0.9280
CMIQ3	AGEQ4	467	-0.16 (-1.38, 1.06)	0.8012
CMIQ4	AGEQ4	387	-0.20 (-1.43, 1.02)	0.7481
P for interaction	0.00028			

Model II**				
Exposure: CMI	Effect modifier: Gender	Statistics		
		N	β(95% CI)	P-values
CMIQ1	male	563	Ref.	
CMIQ2	male	734	-0.02 (-0.13, 0.10)	0.7855
CMIQ3	male	779	-0.09 (-0.20, 0.02)	0.1191
CMIQ4	male	914	0.01 (-0.11, 0.12)	0.9175
CMIQ1	female	984	-0.50 (-1.14, 0.15)	0.1308
CMIQ2	female	813	-0.44 (-1.08, 0.21)	0.1814
CMIQ3	female	768	-0.44 (-1.08, 0.20)	0.1794
CMIQ4	female	633	-0.44 (-1.09, 0.20)	0.1804
P for interaction	0.276			

Model II* adjust for gender, race, alcohol consumption, smoking, diabetes, hypertension, heart disease, hypercholesterolemia, cancer, and the interaction terms for the following variables: gender, race, alcohol consumption, smoking, diabetes, glycohemoglobin, hypertension, heart disease, hypercholesterolemia, cancer.

Model II** adjust for age, race, alcohol consumption, smoking, diabetes, hypertension, heart disease, hypercholesterolemia, cancer, and the interaction terms for the following variables: age, race, alcohol consumption, smoking, diabetes, glycohemoglobin, hypertension, heart disease, hypercholesterolemia, cancer.

**Fig. 2:**
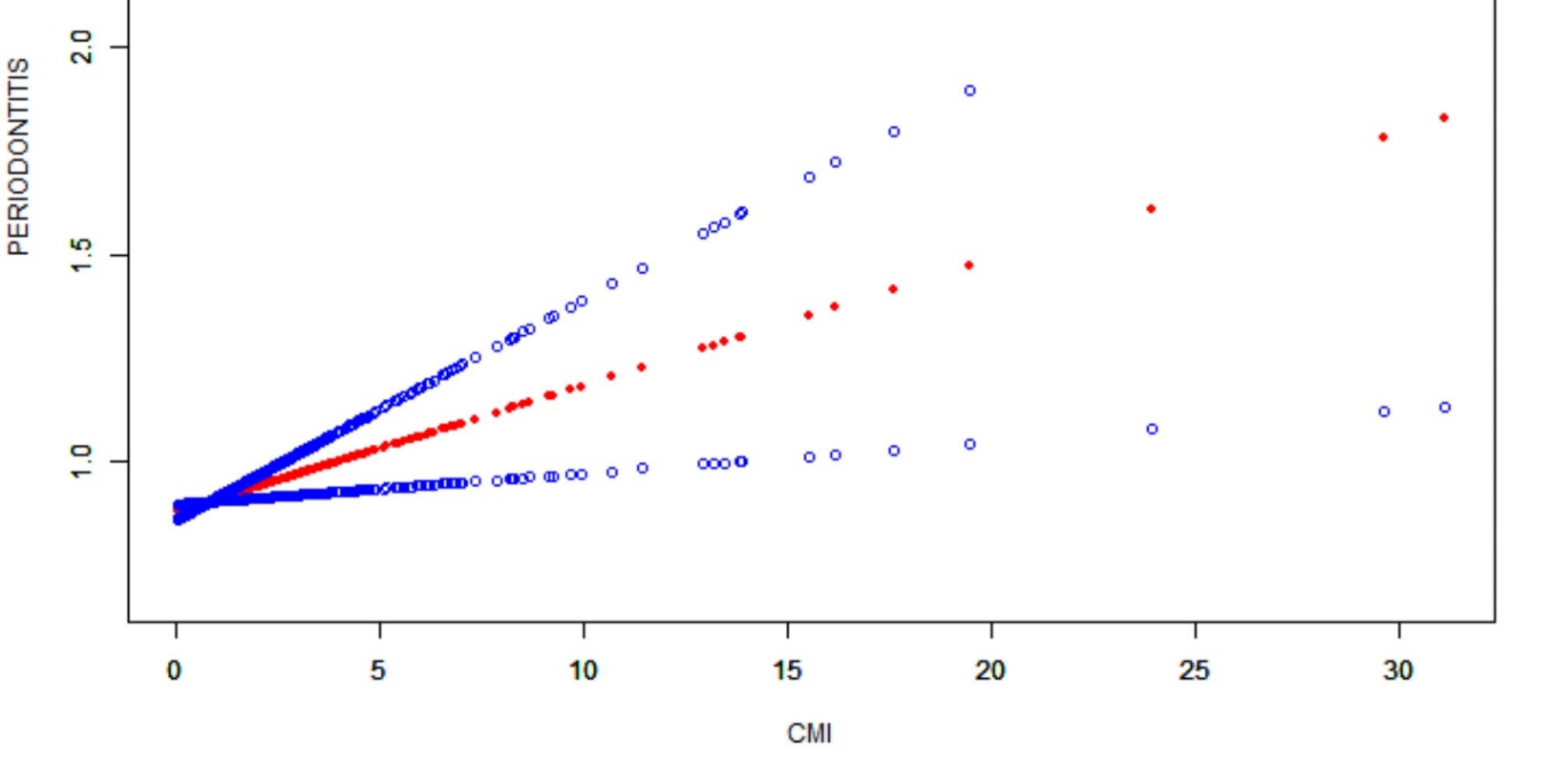
The association between CMI and periodontitis. The solid red line represents the smooth curve fit between variables. Blue bands represent the 95% confidence interval from the fit. CMI, cardiometabolic Index.

**Fig. 3:**
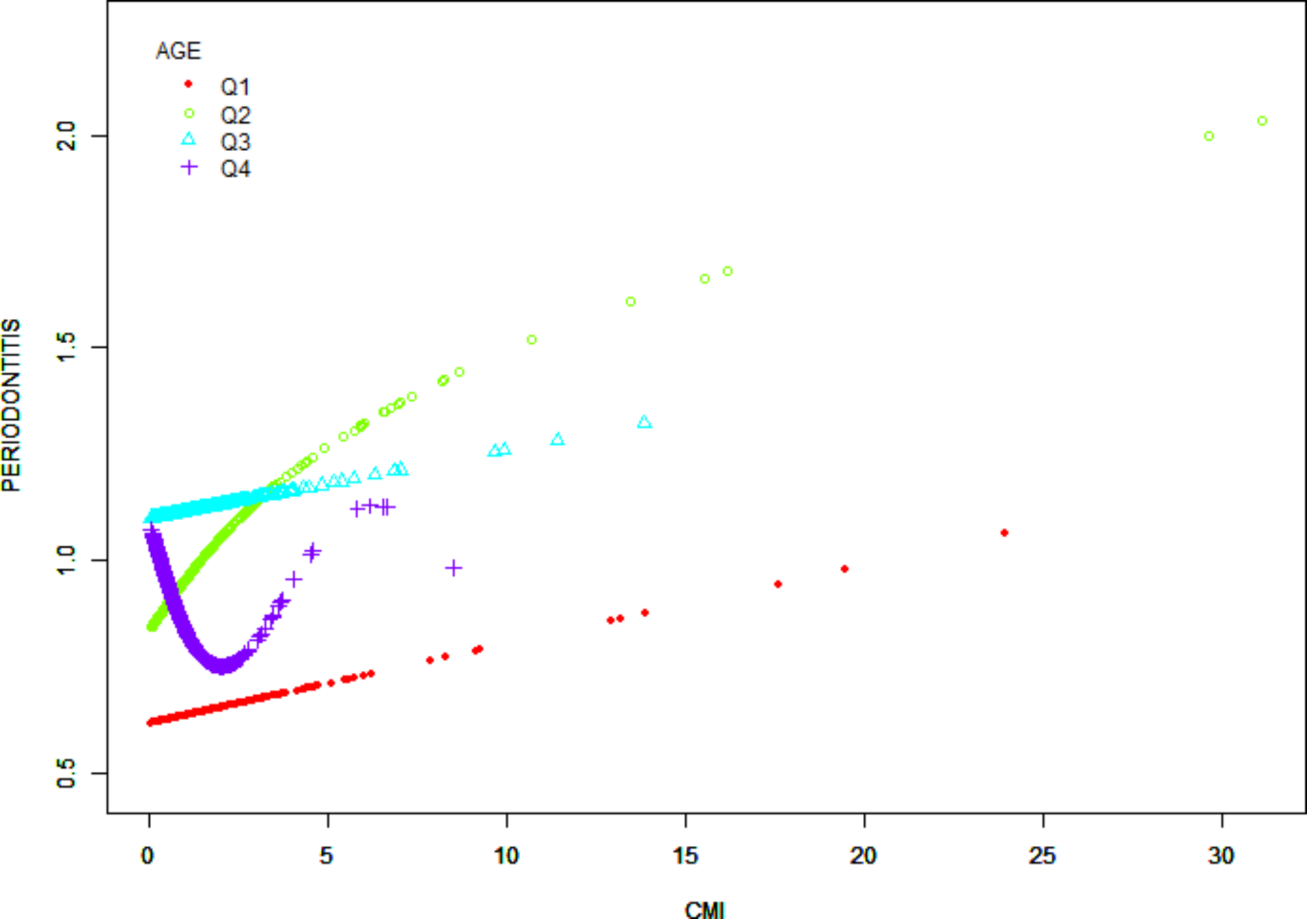
The association between CMI and periodontitis stratified by age. CMI, cardiometabolic Index. Age was segmented into four categories based on quartiles: AGE( > = 30,<41) Q1, AGE( > = 41,<53) Q2, AGE( > = 53,<65) Q3, AGE( > = 65,<=80) Q4.

**Fig. 4:**
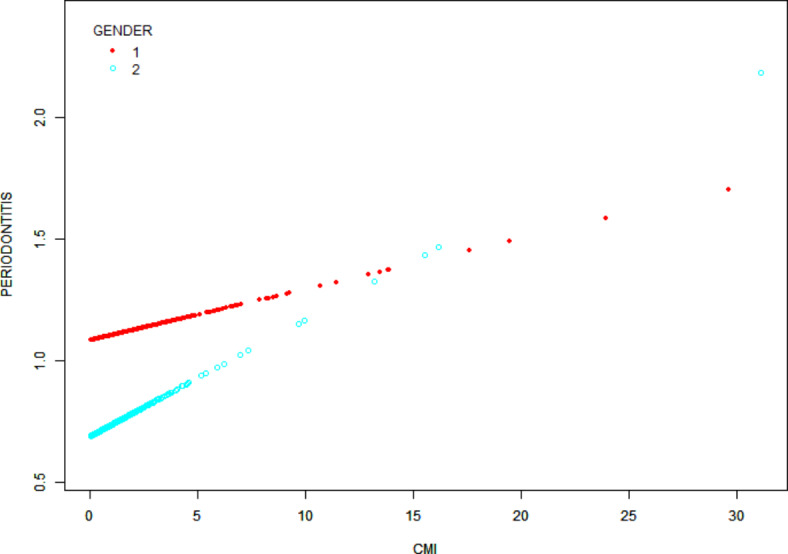
The association between CMI and periodontitis stratified by gender. CMI, cardiometabolic Index. Gender was classified as either male(1) or female(2).

**Fig. 5:**
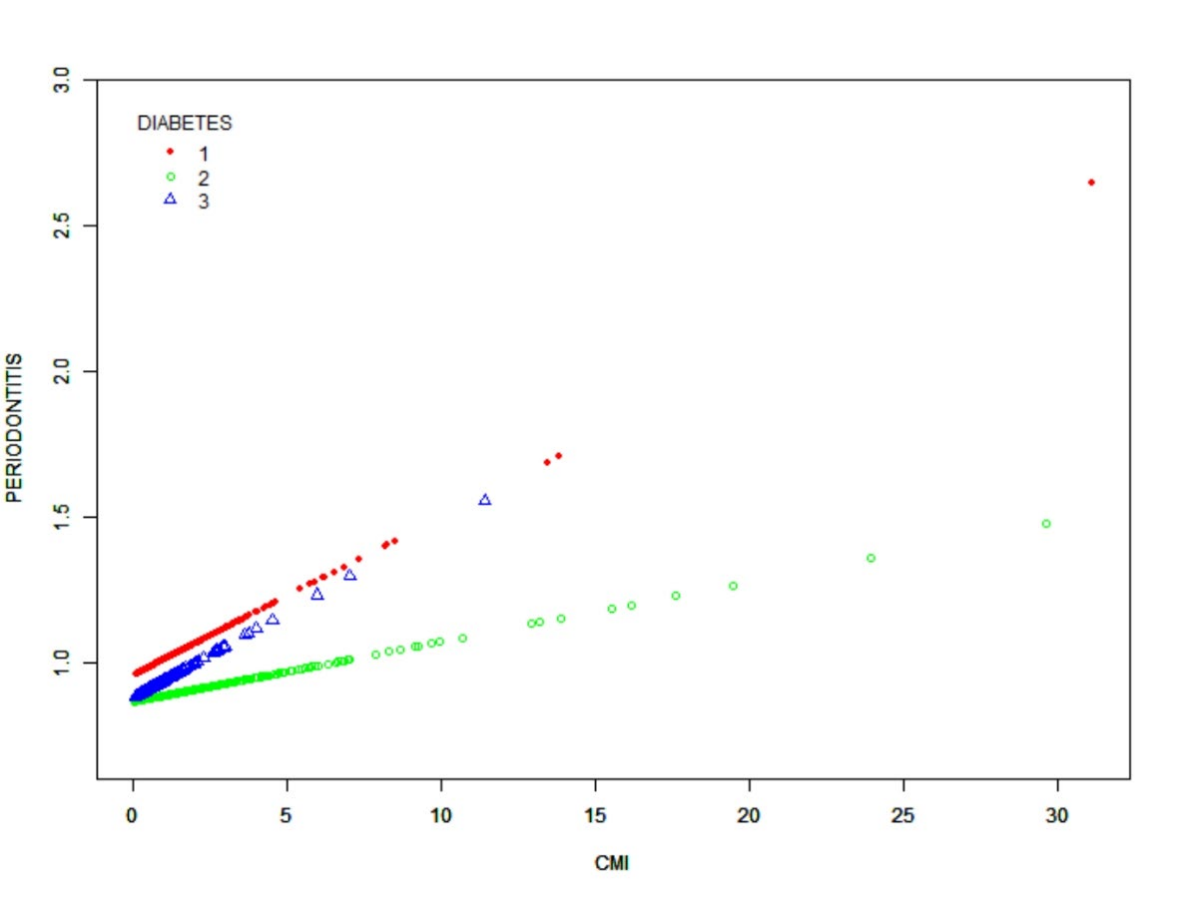
The association between CMI and periodontitis stratified by diabetes. CMI, cardiometabolic Index. Diabetes status was classified into three categories: Yes(1), No(2), or Borderline(3), based on a doctor’s diagnosis.

**Fig. 6:**
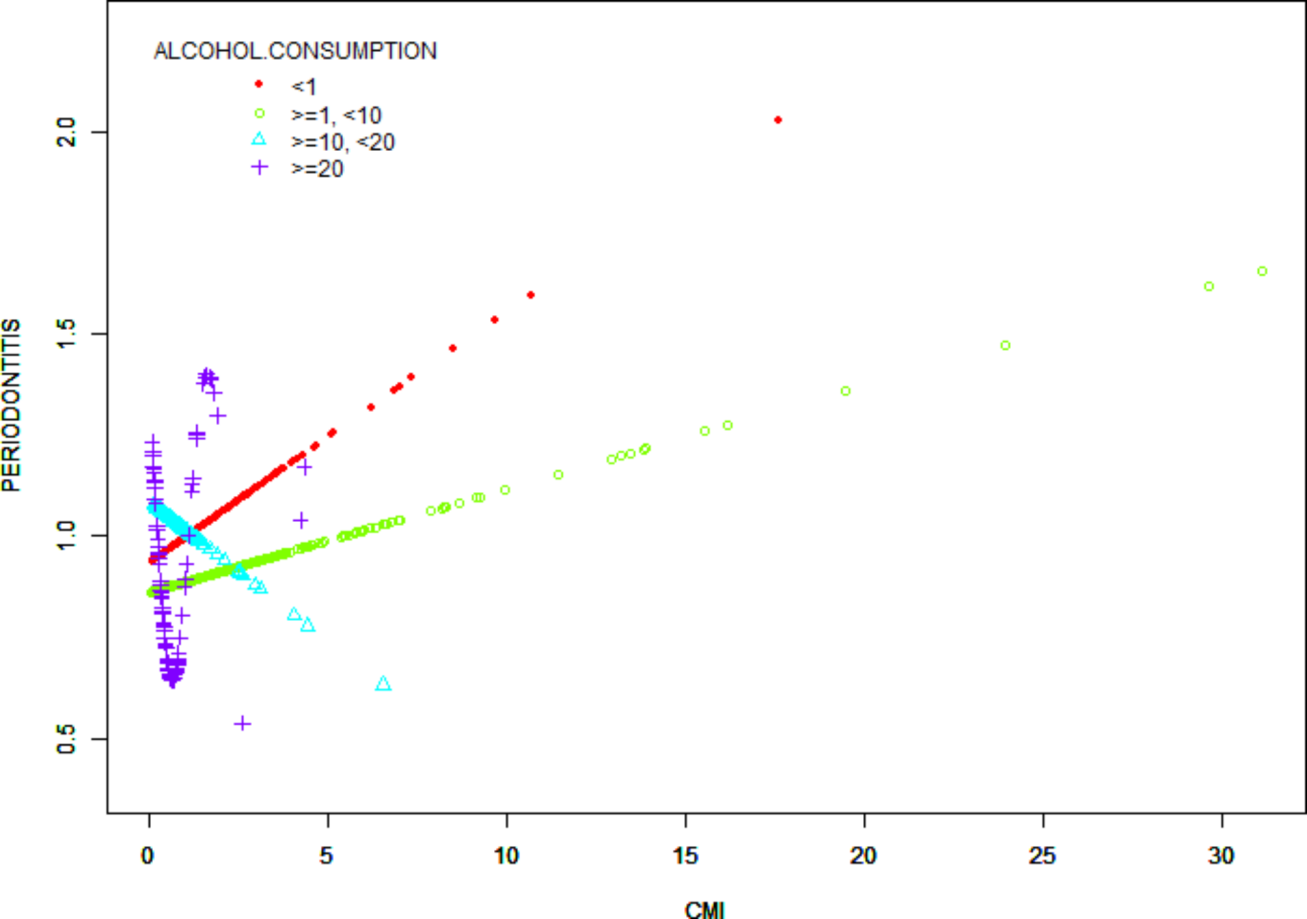
The association between CMI and periodontitis stratified by alcohol consumption. CMI, cardiometabolic Index. Participants were categorized into four groups based on their alcohol consumption frequency over the past 12 months: non-drinkers (< 1), light drinkers ( > = 1, < 10), moderate drinkers ( > = 10, < 20), and heavy drinkers ( > = 20).

Figure 2 shows a smoothed curve analysis of the link between CMI and periodontitis. The analysis is grouped based on age, gender, diabetes, and alcohol consumption, as shown in Figs. 3, 4 and 5, and 6. The smoothed curve fit represents the nonlinear relationship between CMI and periodontitis. Once again, there is a notable and significant link between CMI and periodontitis.

### Relationship between CMI and periodontitis in an elderly population

To explore the relationship between periodontitis and CMI in older individuals, we divided them into four age subgroups. In general, it was noted that the seriousness of periodontitis increased gradually as the CMI levels rose. A special association was found between CMI and periodontitis in the senior population, following a U-shaped pattern, with a folding point of 1.44 (Table 4).

**Table 4:** Analysis of the threshold effect of CMI and periodontitis in different age groups.

AGE	AGEQ1	AGEQ2	AGEQ3	AGEQ4	Total
Model A					
A straight-line effect	0.01 (-0.03, 0.04) 0.6248	0.04 (0.01, 0.08) 0.0106	0.04 (-0.02, 0.10) 0.2090	-0.07 (-0.15, 0.02) 0.1148	0.03 (0.01, 0.05) 0.0164
Model B					
Folding point (K)	0.27	0.64	0.39	1.44	0.17
< K Segment Effect 1	-0.79 (-1.89, 0.31) 0.1579	0.38 (0.07, 0.69) 0.0159	0.36 (-0.40, 1.12) 0.3552	-0.23 (-0.37, -0.08) 0.0022	0.88 (-1.20, 2.97) 0.4071
> K Segment effect 2	0.01 (-0.02, 0.05) 0.4808	0.03 (-0.00, 0.07) 0.0746	0.03 (-0.03, 0.10) 0.3394	0.09 (-0.05, 0.23) 0.2249	0.03 (0.00, 0.05) 0.0207
Difference in effect between 2 and 1	0.81 (-0.30, 1.91) 0.1532	-0.35 (-0.66, -0.03) 0.0324	-0.33 (-1.11, 0.45) 0.4101	0.31 (0.08, 0.55) 0.0084	-0.85 (-2.94, 1.23) 0.4221
Predicted value of the equation at the fold point	0.61 (0.55, 0.67)	1.05 (0.97, 1.12)	1.13 (1.06, 1.21)	0.73 (0.60, 0.85)	0.89 (0.85, 0.92)
Log-likelihood ratio test (p)	0.151	0.031	0.407	0.008	0.421

Results in table: β (95%CI) P-value.

Outcome variable: Periodontitis.

Exposure variable: CMI.

Adjusted variable: gender, race, alcohol consumption, smoking, diabetes, glycohemoglobin, hypertension, heart disease, hypercholesterolemia, cancer.

## Discussion

Until now, there has been no investigation into the correlation between periodontitis and CMI. Periodontitis and several metabolic disorders have inflammation as a shared etiology^16^. This research comprised a total of 6188 individuals. An evident and robust correlation was seen between the severity of periodontitis and the degree of CMI. This connection remained statistically significant even after accounting for potential confounding variables through the use of multivariate logistic regression. Subgroup analysis also revealed a same pattern. Our research indicates that CMI is a reliable indicator for predicting the presence of periodontitis, demonstrating excellent diagnostic accuracy.

Periodontitis is a prevalent global health issue^17^. Periodontitis is a condition where the tissues that support the teeth are gradually destroyed. This is generally identified by clinical attachment loss (CAL), loss of the bone that holds the teeth in place as shown on radiographic examinations or images, the development of pockets around the teeth, and bleeding of the gums^18^. Alveolar bone, periodontal ligament, dentin, and gums are all impacted by the inflammatory disease known as periodontitis^19^. Additionally, it is recognized to elevate the likelihood of systemic inflammation and disease in remote tissues^20^. The research conducted by Hasturk and Sanz identified several potential mechanisms through which periodontitis may impact the distal organs^20,21^. The first mechanism involves the direct migration and colonization of periodontal microorganisms into the distal region, which induces an inflammatory response that is distal to the point of invasion. The second mechanism includes systemic inflammation either by metastatic periodontal inflammation or the activation of soluble inflammatory pathways by periodontal microbes^20,22^ .

Periodontitis has a relationship with metabolic disorders^21,23–27^. CMI serves as a dependable indicator of metabolic disorders^28–31^. Originally employed for the detection of diabetes, CMI exhibited a robust association with gender^7^. A study done by Q.Y. in a Chinese population of middle-aged and older persons revealed a direct relationship between CMI and the likelihood of acquiring new-onset type 2 diabetes mellitus^32^. A high CMI was determined to be a causative element in the onset of type 2 diabetes mellitus within this particular group. There is a correlation between CMI and the likelihood of developing type 2 diabetes mellitus, as demonstrated by numerous investigations^8,9,33^. Multiple inquiries have also substantiated a correlation between CMI and medical illnesses including hyperuricemia, stroke, and obstructive sleep apnea (OSA)^28,34,35^. As the CMI increases, the severity of the metabolic disorder also increases.

The precise mechanism by which CMI impacts the progression of periodontitis is still not fully understood and might be linked to systemic inflammatory reactions. A research conducted by Se. et al. discovered that in persons who are obese and have a high WHtR, an excess of free fatty acids hinders the effectiveness of insulin in regulating glucose metabolism^36^, leading to the development of diabetes mellitus. Dandona et al. discovered that individuals with diabetes and obesity had elevated levels of IL-6 and TNF-α in their bloodstream compared to the general population^37^. IL-6 and TNF-α are essential inflammatory mediators^38,39^; however, the pattern and rate of periodontitis development are determined by the sum of inflammatory responses in systemic tissues^40^. This implies that an elevated CMI is likely to impact the progression of periodontitis.

The latest agreement on periodontal disease highlights the correlation between periodontitis and cardiovascular disease^21^.It is necessary to thoroughly investigate the association between CMI and periodontitis, as there is currently a lack of publications on this topic. The current investigation demonstrated a positive correlation between the severity of periodontitis and the rise in CMI. However, among the older population, there was a curvilinear link between CMI and periodontitis, characterized by a U-shape. The folding point of this relationship was seen at 1.44. The folding point may be due to the following: first, the assessment of periodontitis involves numerous factors, including the probing depth (PD) and clinical attachment loss (AL) of periodontal pockets. Additionally, tooth loss is a severe end-stage manifestation of periodontitis that is most prevalent in older individuals. The current investigation established the degree of periodontitis by evaluating the depth of periodontal pockets (PD) and the amount of clinical attachment loss (AL). When a tooth is removed, the first severe symptoms of periodontitis, including as periodontal disease and attachment loss, are no longer present. The presence of a folding point in CMI does not indicate an improvement in periodontitis; in fact, it may indicate the onset of significant tooth loss. To summarize, the presence of the folding point did not impact the observed strong positive connection between CMI and periodontitis. Additionally, extensive research is needed in the future to further clarify the significance of CMI in periodontitis.

This study is the first to demonstrate a substantial positive relationship between CMI and periodontitis. Furthermore, the sample size is vast and reliable. Nevertheless, this investigation was conducted retrospectively and in a cross-sectional manner, preventing us from evaluating the causal connection between periodontitis and CMI. While efforts were made to account for various confounding factors, there is always a possibility that unaccounted confounders might affect the relationship between periodontitis and CMI.

## Conclusion

To conclude, the presence of periodontitis in the US population is significantly and clearly correlated with an increase in CMI, even after controlling for other variables. The use of CMI as a novel and dependable anthropometric measure may be utilized to accurately forecast the occurrence of periodontitis.

## Data Availability

Publicly available datasets were analyzed in this study. This data can be found here: www.cdc.gov/nchs/nhanes/.
